# Comment on: Benefit of primary and secondary prophylactic implantable cardioverter defibrillator in elderly patients

**DOI:** 10.1002/clc.24224

**Published:** 2024-01-31

**Authors:** Vadim Zakiev, Anna Gvozdeva, Anton Skotnikov

**Affiliations:** ^1^ Russian Clinical and Research Center of Gerontology Pirogov Russian National Research Medical University Moscow Russia; ^2^ Chazov National Medical Research Center of Cardiology Myasnikov Institute of Clinical Cardiology Moscow Russia; ^3^ Department of Medical and Social Assessment, Emergency, and Ambulatory Practice Sechenov First Moscow State Medical University Moscow Russia

To the Editor,

The article by Lewenhardt et al. stated that in primary prevention the proportion of patients who benefited from implantable cardioverter defibrillator (ICD) implantation decreased with increasing age, and there were no patients who benefited from ICD therapy in the group of patients >80 years.^1^ We would like to discuss the possible impact of frailty on the study results.

Frailty is a progressive age‐related deterioration in physiological systems that results in extreme vulnerability to stressors and increases the risk of a range of adverse outcomes including care dependence and death.^2^, ^3^ It is a common geriatric syndrome. The prevalence of frailty in high‐income countries at age 50–64 years is around 4%, increasing to 17% in people older than 65 years.^4^

We suppose that frailty in some patients included in the study may have affected the results and led to negative results in the group of patients >80 years. For example, the recent trial showed that revascularization reduces the primary endpoint (composite of myocardial infarction, need for urgent revascularization, stroke, and death) in patients with non‐ST‐segment elevation acute coronary syndrome (ACS) aged ≥80 years,^5^ while the trial, which included only frail patients, showed no beneficial effect of revascularization.^6^ Finally, frailty is associated with a significantly higher risk of all‐cause mortality and readmission in patients with acute ACS.^7^, ^8^

In our view, subanalysis of the current trial in frail and nonfrail cohorts should be performed if it is possible.

## CONFLICT OF INTEREST STATEMENT

The authors declare no conflict of interest.

## Data Availability

Not applicable.

## References

[1] Lewenhardt M , Kreimer F , Aweimer A , Pflaumbaum A , Mügge A , Gotzmann M . Benefit of primary and secondary prophylactic implantable cardioverter defibrillator in elderly patients. Clin Cardiol. Published online November 14, 2023. 10.1002/clc.24191 PMC10826786 37964443

[2] Fried LP , Tangen CM , Walston J , et al. Frailty in older adults: evidence for a phenotype. J Gerontol A. 2001;56(3):M146‐M157. 10.1093/gerona/56.3.m146 11253156

[3] Clegg A , Young J , Iliffe S , et al. Frailty in elderly people [published correction appears in *Lancet*. 2013;382(9901):1328]. Lancet. 2013;381(9868):752‐762. 10.1016/S0140-6736(12)62167-9 23395245 PMC4098658

[4] Santos‐Eggimann B , Cuenoud P , Spagnoli J , Junod J . Prevalence of frailty in middle‐aged and older community‐dwelling Europeans living in 10 countries. J Gerontol A. 2009;64:675‐681. 10.1093/gerona/glp012 PMC2800805 19276189

[5] Berg ES , Tegn NK , Abdelnoor M , et al. Long‐term outcomes of invasive vs conservative strategies for older patients with non‐ST‐segment elevation acute coronary syndromes. J Am Coll Cardiol. 2023;82(21):2021‐2030. 10.1016/j.jacc.2023.09.809 37968019

[6] Sanchis J , Bueno H , Miñana G , et al. Effect of routine invasive vs conservative strategy in older adults with frailty and non‐ST‐segment elevation acute myocardial infarction: a randomized clinical trial. JAMA Intern Med. 2023;183(5):407‐415. 10.1001/jamainternmed.2023.0047 36877502 PMC9989957

[7] Xu W , Cai Y , Liu H , Fan L , Wu C . Frailty as a predictor of all‐cause mortality and readmission in older patients with acute coronary syndrome: a systematic review and meta‐analysis. Wien Klin Wochenschr. 2020;132(11‐12):301‐309. 10.1007/s00508-020-01650-9 32342196

[8] Bai W , Hao B , Meng W , Qin J , Xu W , Qin L Association between frailty and short‐ and long‐term mortality in patients with critical acute myocardial infarction: results from MIMIC‐IV. Front Cardiovasc Med 2022;9:1056037. 10.3389/fcvm.2022.1056037 36588580 PMC9797732

